# How DNA and RNA Viruses Exploit Host Chaperones to Promote Infection

**DOI:** 10.3390/v13060958

**Published:** 2021-05-21

**Authors:** Kaitlyn Speckhart, Jeffrey M. Williams, Billy Tsai

**Affiliations:** 1 Department of Cell and Developmental Biology, University of Michigan Medical School, 109 Zina Pitcher Place, Room 3043, Ann Arbor, MI 48109, USA; knpugh@umich.edu (K.S.); jefwill@umich.edu (J.M.W.); 2 Cellular and Molecular Biology Program, University of Michigan Medical School, 109 Zina Pitcher Place, BSRB 3043, Ann Arbor, MI 48109, USA

**Keywords:** chaperones, viruses, infection, polyomavirus SV40, human papillomavirus, flavivirus, coronavirus, endoplasmic reticulum, Golgi

## Abstract

To initiate infection, a virus enters a host cell typically via receptor-dependent endocytosis. It then penetrates a subcellular membrane, reaching a destination that supports transcription, translation, and replication of the viral genome. These steps lead to assembly and morphogenesis of the new viral progeny. The mature virus finally exits the host cell to begin the next infection cycle. Strikingly, viruses hijack host molecular chaperones to accomplish these distinct entry steps. Here we highlight how DNA viruses, including polyomavirus and the human papillomavirus, exploit soluble and membrane-associated chaperones to enter a cell, penetrating and escaping an intracellular membrane en route for infection. We also describe the mechanism by which RNA viruses—including flavivirus and coronavirus—co-opt cytosolic and organelle-selective chaperones to promote viral endocytosis, protein biosynthesis, replication, and assembly. These examples underscore the importance of host chaperones during virus infection, potentially revealing novel antiviral strategies to combat virus-induced diseases.

## 1. Background

To cause infection, an incoming viral particle engages a receptor that is expressed on the plasma membrane of the host cell [1]. This interaction leads to receptor-mediated endocytosis, enabling the virus to navigate the densely interconnected endomembrane system within the cell. Navigating through this endomembranous system, which is composed of the endosome, lysosome, Golgi, endoplasmic reticulum (ER), and the nucleus, can result in nonproductive or productive infection. When the virus is delivered to an intracellular compartment where it becomes trapped or is proteolytically degraded, it undergoes nonproductive entry. In contrast, the virus is transported along a productive route when it is targeted along a pathway that allows the viral particle to successfully escape a membranous compartment, enabling it to reach an intracellular destination that supports transcription, translation, and replication of the viral genome. These events, in turn, result in the assembly and morphogenesis of the new viral progeny. In the final step, the mature viral particle exits the host cell to initiate the next round of infection. Indeed, the strategies exploited by viruses to accomplish each of these entry steps—endocytosis, membrane penetration, viral genome expression and replication, as well as assembly and egress—as part of a choreographed program to achieve productive infection are unique and highly varied.

Despite these differences, one emerging common principle is that viruses co-opt host molecular chaperones to promote distinct entry steps. Molecular chaperones typically function to fold or unfold a protein, helping to achieve its native conformational state or to untangle its structural configuration [2]. Molecular chaperones also assist in the assembly or disassembly of macromolecular structures. Importantly, the intrinsic activities of molecular chaperones can be hijacked by viruses during infection. From the initial viral entry process to the final egress event, cellular chaperones have been documented to play decisive roles in governing these steps. In this review, we describe how viruses across different families exploit the activities of soluble, membrane-bound, and organelle-specific host chaperones to accomplish the select entry steps required for successful infection.

## 2. Exploiting ER and Cytosolic Chaperones during ER Escape and Disassembly of Polyomavirus

Polyomaviruses (PyVs) cause devastating human diseases, particularly in immunocompromised individuals [3]. Well-studied human PyVs include the BK PyV that triggers hemorrhagic cystitis and nephropathy, JC PyV which is responsible for progressive multifocal leukoencephalopathy, and the Merkel cell PyV which is the causative agent of Merkel cell carcinoma [4]. Simian virus 40 (SV40) represents the prototype PyV, displaying genetic and structural similarities to human PyVs, as well as using a comparable infection pathway as its human counterparts [5]. Predictably, insights into the entry mechanism of SV40 have informed the cellular basis of human PyV infection. 

**Figure 1:**
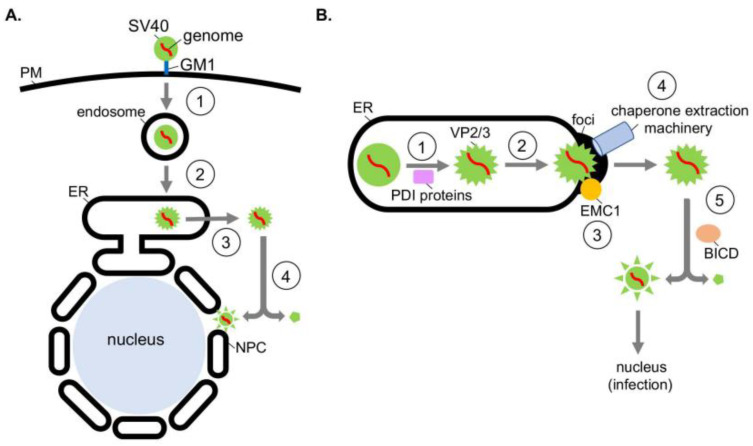
Exploiting ER and cytosolic chaperones during ER escape and disassembly of polyomavirus SV40. (**A**) Polyomavirus SV40 entry pathway. During entry, SV40 undergoes receptor-mediated endocytosis, trafficking to the endosome and then the ER. Here it penetrates the ER membrane to reach the cytosol and then the nucleus to cause infection. (**B**) In the ER, PDI family proteins (PDI, ERp57, and ERdj5) reduce and isomerize the SV40 disulfide bonds **(step 1)**, while another PDI family member (ERp29) unfolds the VP1 C-terminal arm—these reactions disrupt the viral architecture that exposes the SV40 hydrophobic VP2 and VP3 proteins. As a consequence, a hydrophobic SV40 particle is formed which integrates into the ER membrane **(step 2)**. SV40 then reorganizes the ER membrane to construct an exit site (called foci) where the virus crosses to reach the cytosol. During foci formation, SV40 directs the ER membrane protein EMC1 to relocate to the foci where it stabilizes the membrane-inserted SV40 **(step 3)**. SV40 further triggers DNA J protein family members (B12, B14, and C18) to accumulate in the foci—this recruits a cytosol-localized chaperone extraction machinery (Hsc70, Hsp105, Bag2, and SGTA) that propels SV40 into the cytosol **(step 4)**. The dynein motor adaptor BICD disassembles the cytosol-localized virus **(step 5)**, forming a subviral particle that enters the nucleus to trigger infection.

SV40 is composed of 72 pentamers of the VP1 coat protein that encloses its 5 kilobase-pair double-stranded DNA genome [6,7]; each VP1 pentamer contains either a VP2 or VP3 internal hydrophobic structural protein [8]. The native viral particle has a diameter of 50 nm when properly assembled. To initiate infection, SV40 interacts with the glycolipid GM1 ganglioside receptor at the plasma membrane [9], triggering receptor-mediated endocytosis that delivers the virus first to the endosome (Figure 1A, step 1) [10] and then the ER (Figure 1A, step 2) [11]. From this compartment, SV40 penetrates the ER membrane and escapes into the cytosol (Figure 1A, step 3) [12,13]. The virus disassembles in the cytosol and is then transported into the nucleus (Figure 1A, step 4) [12,14,15] where transcription and replication of the viral genome ensue, resulting in lytic infection or cellular transformation. 

Recent studies on the mechanism of the SV40 ER escape and ensuing cytosol-dependent disassembly have revealed a network of ER-associated and cytosolic chaperones as responsible for these processes. Specifically, upon reaching the ER lumen from the cell surface, ER luminal redox factors belonging to the protein disulfide isomerase (PDI) family reduce and isomerize the disulfide bonds of the viral particle (Figure 1B, step 1) [16,17,18]. Because these covalent bonds stabilize the SV40 architecture, disulfide bond reduction and isomerization destabilize the virus. In the case of the murine PyV, another PDI family member (called ERp29) exerts its chaperone activity, locally unfolding the VP1 C-terminal arm that provides inter-pentamer support of the virus [19]—this action further disrupts the overall viral structure. The cumulative effect of these destabilizing events is exposure of the viral hydrophobic VP2 and VP3 proteins [13,20,21]. As a result, a hydrophobic SV40 particle is generated in the ER lumen, which then inserts into the ER lipid bilayer (Figure 1B, step 2) [19,21].

Once SV40 has integrated into the ER membrane, it reorganizes the ER to form an exit site (called foci) from which it gains access to the cytosol [13,22,23,24]. During formation of the foci, SV40 reorganizes specific ER membrane proteins into the foci structure to assist in viral escape. For instance, the ER membrane protein EMC1, a key component of the multi-subunit ER membrane complex (EMC), relocates to the foci where it acts as a transmembrane chaperone, stabilizing the membrane-embedded SV40 (Figure 1B, step 3) [25]. This prevents premature disassembly of the viral particle, thereby ensuring proper membrane penetration. 

In parallel, SV40 induces several DNA J protein family members (called B12, B14, and C18) to accrue in the foci [22,23]. A J protein contains a J-domain motif that binds to and stimulates the ATPase activity of Hsc70 chaperone [26]. J protein accumulation at the ER-foci subsequently recruits a cytosol-localized chaperone extraction machinery (comprising of Hsc70, the nucleotide exchange factors Hsp105 and Bag2, and the co-chaperone SGTA) that ejects SV40 from the ER into the cytosol (Figure 1B, step 4) [22,23,27,28]. Mechanistically, this is accomplished when the chaperone extraction machinery—via an iterative ATPase-dependent binding-and-release cycle—ratchets SV40 into the cytosol to complete the membrane escape process. Although the cytosolic Ubiquilin4 (Ubqln4) chaperone further assists in SV40 ER escape, its precise role remains unclear [29]. 

Upon reaching the cytosol, the SV40 capsid proteins are partially disassembled by the cytosolic dynein motor adaptor called BICD, which surrounds the ER-foci (Figure 1B, step 5) [15]. This reaction generates a subviral particle that is competent to enter the nucleus to cause infection. BICD-dependent viral disassembly is unexpected because this activity has not been reported for any dynein motor adaptors. In summary, the combination of ER soluble and membrane chaperones, along with an ER-associated cytosolic extraction machinery and a motor adaptor possessing an unanticipated disassembly activity, promote viral ER-to-cytosol escape followed by capsid disassembly to enable successful infection of PyV SV40.

## 3. Co-Opting Soluble and Membrane Chaperones during Internalization, Disassembly, and Endosomal Membrane Insertion of Human Papillomavirus

In addition to PyV, the human papillomavirus (HPV) is yet another DNA tumor virus that co-opts host chaperones during infectious entry. HPV is a highly prevalent virus, infecting approximately 80 million adults in the United States [30]. It belongs to a large family of viruses, with each type categorized as either low- or high-risk. Persistent infections with high-risk HPV types cause a multitude of cancers, and are the primary cause of cervical, anogenital, as well as oropharyngeal cancer [31]. 

**Figure 2:**
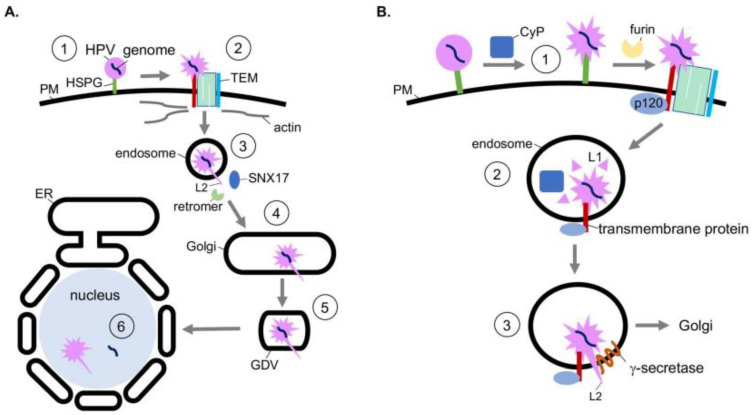
Co-opting soluble and transmembrane chaperones during internalization, disassembly, and endosomal membrane insertion of human papillomavirus. (**A**) HPV entry pathway. During entry, HPV undergoes receptor-dependent endocytosis, transporting to the endosome and then the Golgi. HPV then buds from the Golgi during mitosis, entering the nucleus after nuclear membrane breakdown to cause infection. (**B**) At the plasma membrane, the peptidyl-prolyl isomerase activity of cyclophilins (CyP) induces conformational changes in the viral capsid proteins that expose a furin cleavage site at the N-terminus of L2 (step 1). After furin cleavage, the virus is transferred to the tetraspanin-enriched microdomain (TEM) and endocytosed. In the endosome, cyclophilins mediate partial dissociation of L1 from L2 and the viral genome (step 2), further exposing L2. Additionally, in the endosome, the transmembrane protein γ-secretase interacts with L2 and acts as a chaperone to promote the insertion of the L2 C-terminus across the endosomal membrane (step 3). The cytosol-exposed L2 recruits host sorting factors, which traffic the virus to the Golgi en route for infection.

Structurally, HPV is a nonenveloped double-stranded DNA tumor virus composed of 72 pentamers of the major capsid protein L1. The L1 pentamers encase up to 72 copies of the minor capsid protein L2. Together, these encapsulate the 8 kilobase-pair double-stranded DNA genome [32]. The assembled virion has a diameter of 55 nm [33]. HPV infects mitotically active, basal epithelial cells by the initial binding of L1 to heparin sulfate proteoglycans (HSPGs) on the cell surface (Figure 2A, step 1) [34,35,36,37]. A series of conformational changes exposes the N-terminus of L2. L2 is then cleaved by the furin protease [38] and the viral particle is transferred to a tetraspanin-enriched microdomain (TEM) composed of tetraspanins CD151 and CD63, integrins α6 and β4, annexin A2, and an unidentified secondary receptor (Figure 2A, step 2) [39,40,41,42,43]. Next HPV is asynchronously endocytosed in a clathrin-, caveolin-, and dynamin-independent manner. Viral internalization is instead connected to actin polymerization [44,45]. 

After delivery to the endosome, the viral capsid proteins begin to dissociate from the viral genome in a pH-dependent manner. This disassembly is partial, as L2 and a subset of L1 remains associated with the viral genome [46,47]. Post-disassembly, L2 is inserted into the endosomal membrane, resulting in exposure of the L2 C-terminus to the cytosol, which in turn recruits host sorting factors such as the retromer complex and sorting-nexin 17 (SNX17) (Figure 2A, step 3) [48,49,50]. The virus is then trafficked to the Golgi, where it remains until the onset of mitosis (Figure 2A, step 4). Upon mitosis, Golgi fragmentation generates Golgi-derived vesicles (GDVs) harboring the virus (Figure 2A, step 5) [51]. The HPV-harboring GDVs enter the nucleus during mitotic nuclear envelope breakdown and associate with the host cell’s condensed chromosomes [52,53]. After nuclear envelope reformation, L2 and the viral genome penetrate the vesicle membrane through an unknown mechanism, enabling transcription and replication of the viral genome (Figure 2A, step 6) [54]. Whether L2 is released from the viral genome in this process remains unclear.

The action of host chaperones regulates the earliest stage of HPV entry. Specifically, conformational changes of the viral capsid at the plasma membrane are mediated in part by the peptidyl-prolyl isomerase activity of cyclophilin (CyP), specifically cyclophilin B [55]. These chaperones are localized in the extracellular milieu and lumen of endosomal compartments. Prior to internalization, the conformational changes induced by the CyP B isomerase activity at the cell surface is thought to expose the L2 N-terminus from the L1 pentamers (Figure 2B, step 1) [55]. Importantly, the exposed N-terminus harbors a furin cleavage site. Although furin cleavage of L2 can occur without the chaperone activity of cyclophilins, viral uptake is accelerated after cyclophilin activity [55]. Evidence also suggests CyPs contribute to capsid disassembly in the endosome after internalization [56]. Specifically, subsequent to endosome acidification, cyclophilins mediate the partial dissociation of L1 from L2 and the viral genome (Figure 2B, step 2) [56]. Loss of this activity inhibits the ability of HPV to reach the nucleus. Thus, the chaperone activity of cyclophilins at two distinct entry steps promotes productive HPV infection. 

After the HPV particle is partially disassembled in the endosome, L2 is inserted into the endosomal membrane resulting in exposure of L2 to the cytosol. This is a decisive step because the cytosol-exposed L2 engages the host sorting factors which target the virus along a productive infection pathway. Topologically, when L2 is inserted into the endosomal membrane, its N-terminus remains associated with the viral genome while the C-terminus is exposed to the cytosol [57]. Strikingly, membrane insertion of L2 relies on the chaperone activity of the host transmembrane protein γ-secretase [58]. γ-secretase, a protein complex with four subunits, typically cleaves transmembrane proteins [59]. Intriguingly, the protease activity of γ-secretase is not required for HPV infection [58]. Instead, HPV hijacks a novel chaperone function of γ-secretase. In this case, γ-secretase directly binds to HPV L2, promoting insertion of L2 across the endosomal membrane (Figure 2B, step 3). This insertion event is further enabled by the presence of a cationic cell-penetrating peptide sequence on the C-terminus of L2 [60].

The delivery of HPV to γ-secretase in the endosome relies on the cytosolic γ-secretase adaptor p120 catenin, which normally targets cellular transmembrane proteins to γ-secretase [61,62,63]. In this scenario, HPV is thought to be delivered to γ-secretase when p120 targets an unidentified transmembrane protein—which is in complex with HPV—to γ-secretase. Not surprisingly, the mutation of γ-secretase that disrupts γ-secretase-p120 binding prevents HPV infection [61]. Thus, HPV co-opts host soluble and transmembrane chaperones that facilitate viral internalization, disassembly, and endosomal membrane insertion to cause infection.

## 4. Hijacking Cytosolic and ER-Localized Chaperones to Promote Flavivirus Infection

In addition to the double-stranded DNA tumor viruses PyV and HPV, single-stranded RNA viruses—including flaviviruses and coronaviruses—also exploit the intrinsic activities of host molecular chaperones to support infection. Within the flavivirus family, its prominent members include Dengue virus (DENV), Zika virus (ZIKV), Japanese encephalitis virus (JEV), West Nile virus (WNV), yellow fever virus (YFV), and tick-borne encephalitis virus (TBEV). These viruses are among the most crucial arthropod-borne viruses that cause a myriad of human diseases [64]. 

**Figure 3:**
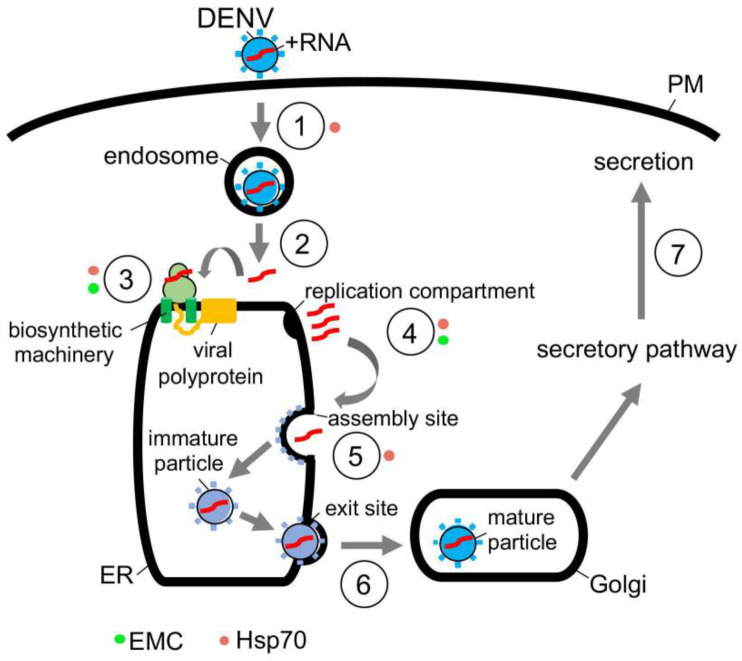
Hijacking cytosolic and ER-localized chaperones to promote flavivirus infection. Flavivirus DENV entry pathway (see text for more details). Hsp70 chaperone, in concert with J proteins, facilitate virus endocytosis, protein biosynthesis, and assembly. Hsp70 is proposed to mediate endocytosis of the incoming DENV particle (**step 1**, **orange circle**), but how this is accomplished remains unclear. In contrast, Hsp70 binds directly to the NS5 RNA polymerase in order to promote biosynthesis and function of this enzyme (**step 3, orange circle**), thereby indirectly facilitating viral replication (**step 4, orange circle**). Hsp70 also interacts with and stabilizes the C protein to support viral assembly (**step 5, orange circle**). In addition to cytosolic Hsp70, the ER membrane protein complex EMC likewise contributes to DENV infection by assisting in biosynthesis of the nonstructural membrane proteins NS4A and NS4B (**step 3, green circle**). Since NS4A and NS4B are important for formation of the virus replication compartment (**step 4, green circle**), EMC thus supports virus replication indirectly.

In the case of DENV, this flavivirus initiates infection by interacting with a receptor on the cell surface that leads to internalization (Figure 3, step 1) [65]. Once the viral particle reaches the endosome, the low pH environment induces fusion of the viral and the endosomal membranes, thereby releasing the viral nucleocapsid into the cytosol where it is uncoated (Figure 3, step 2) [66]. Upon uncoating, the positive-sense RNA genome is targeted to the ER where it undergoes co-translational translocation by using the biosynthetic machinery, generating a single viral polyprotein (Figure 3, step 3) [66]. Viral and host proteases next cleave the polyprotein, resulting in formation of three structural proteins (C, prM and E) and seven nonstructural proteins (NS1, NS2A, NS2B, NS3, NS4A, NS4B and NS5) [67]. While the structural proteins become physical components of the new viral progeny, many of the nonstructural proteins play crucial roles in facilitating viral replication, in part, by manipulating the ER structure [68]. These manipulations produce different ER-derived structures that in turn support replication of the viral genome (Figure 3, step 4) [68,69]. 

Once the replication compartment is formed and replication of the viral RNA genome is completed, the new viral progeny begins to assemble (Figure 3, step 5) [70,71]. This is initiated when the viral genomic RNA and C protein merge to become the nucleocapsid—the nucleocapsid then buds into the ER lumen at an assembly site, recruiting the prM and E membrane proteins in the process. The assembled immature viral particle subsequently leaves the ER via an exit site, trafficking to the Golgi where it undergoes further maturation (Figure 3, step 6) [69,70]. Finally, the mature virus exits the host cell via transport along the anterograde pathway en route for secretion (Figure 3, step 7). 

The actions of molecular chaperones have been reported to promote many of these distinct DENV entry steps. For instance, cytosolic Hsp70 chaperone, in concert with dedicated DNA J proteins, is thought to facilitate viral endocytosis, protein biosynthesis, and assembly [71]. Mechanistically, how Hsp70 mediates endocytosis of the incoming DENV particle remains unclear to date. However, Hsp70 can directly act on NS5 [71]—an RNA polymerase needed for replication of the RNA genome—to promote both the biosynthesis and activity of this enzyme (Figure 3, step 3); in this manner, Hsp70 indirectly supports viral replication (Figure 3, step 4) [71]. Hsp70 has also been shown to bind to and stabilize the C protein of DENV to promote viral assembly (Figure 3, step 5) [71]. Likewise, Hsp70 participates in the endocytosis, RNA genome replication, and viral assembly of the related ZIKV [72], presumably by imparting a similar mechanism as in DENV infection. In addition to Hsp70, other cytosolic chaperones including Hsp90 [73,74] and the TriC/CCT complex [75,76] have also been implicated in flavivirus infection, although a better understanding of their precise roles is needed. 

Along with cytosolic chaperones, ER-associated molecular chaperones also play critical functions during flavivirus infection. For instance, the ER-resident EMC transmembrane chaperone contributes to DENV and ZIKV infection. EMC does so by promoting the biogenesis of DENV and ZIKV multipass membrane proteins NS4A and NS4B (Figure 3, step 3) [77,78,79]. How might the EMC selectively target only NS4A and NS4B amongst all the viral nonstructural proteins? One unique characteristic of NS4A and NS4B is that both proteins harbor two marginally hydrophobic transmembrane segments—this property makes these segments difficult to insert into the ER membrane during biosynthesis. For this reason, the chaperone function of the EMC is exploited to ensure the proper membrane insertion of NS4A and NS4B within the ER lipid bilayer. Because NS4A and NS4B exert critical roles in generating the aforementioned flavivirus replication compartments (Figure 3, step 4) [80,81,82], EMC therefore supports virus replication, albeit indirectly. Another ER-resident chaperone called BiP was posited to regulate flavivirus infection [83], although the molecular basis of the BiP’s action during viral entry is not entirely obvious. In sum, the emerging picture is that both cytosolic and ER-localized chaperones are exploited to support different stages of the flavivirus-infection life cycle.

## 5. Commandeering ER-Associated Chaperones during Coronavirus Entry

Coronaviruses (CoVs) are enveloped, positive-sense, single-stranded RNA viruses. They are part of the subfamily *Coronaviridae* which contains four distinct subgroups: alpha, beta, gamma, and delta CoV, with the alpha and beta subgroups being the most prevalent. CoV infections in humans typically lead to mild symptoms such as the common cold, including infections caused by human coronavirus OC43 (HCoV-OC43), human coronavirus HKU1 (HCoV-HKU1), and human coronavirus 229E5 (HCoV-229E5) [84]. However, highly pathogenic beta CoVs have emerged, resulting in significant illness and death in humans. The Middle East Respiratory Syndrome-related coronavirus (MERS-CoV) outbreak, beginning in 2012, has led to over 2500 confirmed cases with a remarkable 36% death rate [85]. Additionally, Severe Acute Respiratory-related virus (SARS-CoV) has caused over 8000 confirmed cases leading to over 750 deaths [86]. Most recently and notably has been the emergence of SARS-CoV-2, the causative agent of coronavirus disease 2019 (COVID-19), which has led to a global pandemic that continues. To date, there are over 137 million confirmed cases of COVID-19 which have led to nearly 3 million deaths in humans [87]. Thus, the need to gain further understanding of these viruses and how they exploit host cell factors cannot be overstated. 

**Figure 4:**
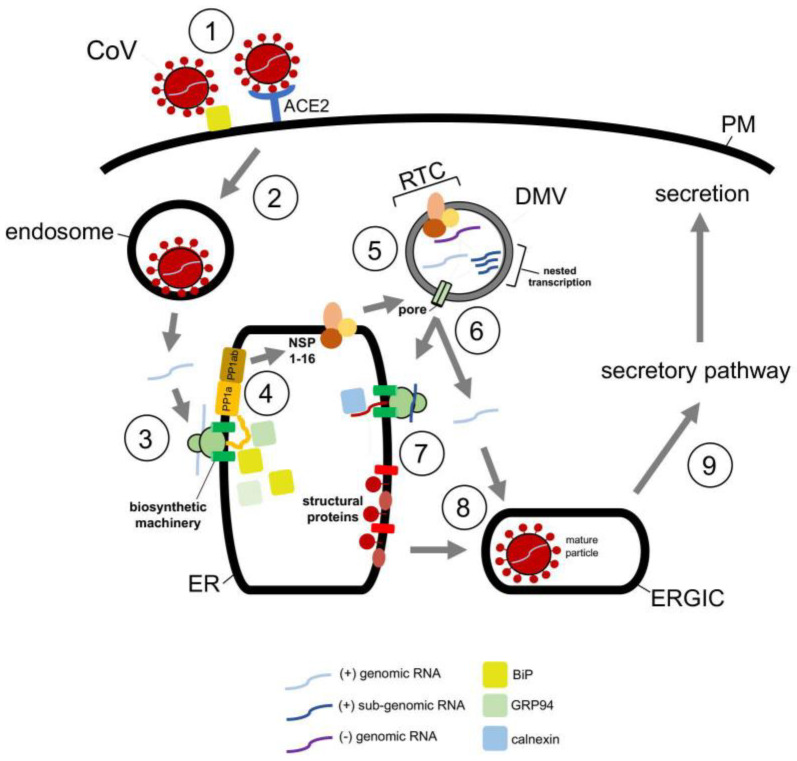
Repurposing ER-associated chaperones during coronavirus entry. Coronavirus CoV entry pathway (see text for more details). Initial attachment to the plasma membrane and subsequent receptor binding to the viral particle at the cell surface is facilitated by the ER luminal BiP chaperone **(step 1)**. The virus then undergoes endocytosis to reach the endosome **(step 2).** Here fusion between the viral and endosomal membranes results in release of the viral genome into the cytosol. The cytosol-localized genomic RNA is then cotranslationally translocated on the ER membrane **(step 3)**, generating the viral PP1a and PP1ab polypeptides **(step 4)**; in this step, the ER-resident chaperones BiP and GRP94 are thought to facilitate proper protein folding. The polyproteins are proteolytically cleaved, forming NSP1-16. Some of these nonstructural proteins (NSPs) assist in formation of the double-membrane vesicle (DMV). Within the DMV, the replicase and transcriptase complex (RTC) is formed **(step 5)**. At this site, viral genome replication occurs, producing many copies of the full-length RNA genome. Additionally, subgenomic RNAs are generated via nested transcription. The full-length and subgenomic RNAs are released from the DMV **(step 6)**. Those subgenomic RNAs encoding M, S, and E are delivered to the ER where these structural proteins are synthesized **(step 7)**; in this process, the ER luminal calnexin chaperone assists in proper folding of the viral proteins. By contrast, the subgenomic RNA encoding N is translated in the cytosol to form the N protein which complexes with the newly-synthesized viral full-length genomic RNA. The M, S, and E structural proteins exit the ER and transport to the ERGIC for packaging with the N protein-genomic RNA complex **(step 8)**. Finally, the mature viral particle is formed and trafficked along the secretory pathway for secretion **(step 9)**.

Structurally, CoVs are approximately 100 nm in diameter with 5′-capped genomes that range between 26 to 32 kb—the largest of all RNA virus genomes [88]. Predictably, beta CoVs share a similar and complicated replication life cycle. SARS-CoV and SARS-CoV-2 cellular entry is initiated by binding of the viral spike (S) protein to the same cell surface receptor, angiotensin converting enzyme 2 (ACE2) (Figure 4, step 1) [89]. This binding event triggers endocytosis of the viral particle which targets the virus to the endosome in a clathrin-/caveolae-independent manner (Figure 4, step 2) [90]. The viral nucleocapsid is subsequently released into the cytosol after fusion of the viral and endosomal membranes [91].

Upon release into the cytosol, the ER then assumes a key role during the replication cycle of CoVs. The viral RNA genome engages the ER membrane, where it is thought to undergo cotranslational translocation (Figure 4, step 3). The product of this step is a set of membrane-embedded polyproteins, PPa1 and PP1ab (Figure 4, step 4) [92,93]. These polypeptides encode the nonstructural proteins (NSP1-16) of which NSP3, NSP4, and NSP6 trigger formation of ER-derived double-membrane vesicles (DMVs) and convoluted membranes (CVs) that will be the sites of the replicase and transcriptase complex (RTC) (Figure 4, step 5) [94,95,96,97,98]. At the RTC, viral genome replication occurs, generating many copies of the full-length RNA genome. Additionally, via nested transcription, subgenomic RNAs—which encode the spike (S), nucleocapsid (N), membrane (M), and envelope (E) structural proteins—of CoVs are made [94,95,96,97].

The subgenomic RNAs encoding S, M, and E are ejected from the DMV via an NSP3-containing pore that spans the double-membrane of the DMV (Figure 4, step 6), and are subsequently delivered to the ER where they are translated into the structural components of CoVs (Figure 4, step 7) [99,100]. Although the subgenomic RNA encoding the N protein likely uses the same escape route, it is instead synthesized in the cytosol. Newly synthesized S, M, and E are then targeted to the ER−Golgi intermediate complex (ERGIC) where they assemble with the N protein (Figure 4, step 8), packaging into new viral progeny that are transported along the secretory pathway for eventual secretion (Figure 4, step 9) [100,101,102,103].

Given that the ER executes a key function in CoV infection, it is perhaps not surprising that ER-associated chaperones promote distinct viral entry steps. In fact, ER chaperones have been reported to facilitate the earliest step of viral entry. For instance, using gain- and loss-of-function approaches, the spike protein of both MERS-CoV and the bCoV-HKU9 beta coronavirus was found to engage BiP at the cell surface (Figure 4, step 1) [104,105]; BiP is normally an ER luminal chaperone that mediates protein folding and degradation pathways within the ER [106]. However, in the case of both MERS-CoV and bCoV-HKU9, BiP assists the cellular uptake of these viruses. This is accomplished when BiP exits the ER to reach the cell surface where it binds to the S protein of the viral particle, thereby facilitating subsequent interaction of the S protein to the dipeptidyl peptidase 4 (DPP4) cell surface receptor essential for viral entry [104,106]. 

At the ER, CoVs commandeer the activities of ER-associated chaperones, which are generated through virally-induced ER stress. SARS-CoV, for example, triggers the unfolded protein response (UPR) via its S protein [107,108]. The UPR is a highly conserved ER-dependent stress response designed to respond to stress caused by accumulation of misfolded or non-native proteins in the ER. This ER stress can activate three independent branches of the UPR that serve to attenuate overall protein translation, increase protein degradation, and upregulate expression of ER chaperones which in turn assist the folding of misfolded clients [109]. In the case of SARS-CoV, the virus activates the PERK ER transmembrane protein, representing one branch of the UPR [110]. This leads to phosphorylation of eIF2α, which in turn lowers the overall translation while upregulating the production of ER-resident folding chaperones GRP94 and BiP. In principle, enhanced GRP94 and BiP production can assist folding of the viral structural (Figure 4, step 7) and nonstructural (Figure 4, step 4) proteins that are essential for productive infection. Downstream of this event, SARS-CoV structural proteins E, M, and S have been shown to bind to calnexin (Figure 4, step 7), an ER-resident lectin chaperone that is part of a profolding complex [111]. This engagement ensures proper folding of the structural components before exiting the ER en route to the ERGIC for assembly (Figure 4, step 8).

The importance of ER-associated chaperones during CoV infection has been reignited by COVID-19. Indeed, the global effort to identify host proteins exploited by SARS-CoV-2 that promote infection has uncovered a myriad of ER-dependent chaperones, including EDEM3, ERLEC1, RTN4, Sigma-1 receptor, that play essential (but poorly characterized) roles during SARS-CoV-2 entry [112,113]. Without a doubt, a united effort is urgently needed to illuminate how these ER chaperones support SARS-CoV-2 infection, with the anticipation that these insights will reveal new antiviral strategies to blunt the current COVID-19 pandemic.

## 6. Conclusions

Exploiting host chaperones is a common strategy used during the entry of many viruses. As described in this review, both DNA and RNA viruses take advantage of host chaperones in a variety of ways to ensure a productive viral life cycle. The chaperone functions hijacked by these viruses are used to support different entry steps, including cell surface attachment and endocytosis, unfolding and disassembly, as well as membrane penetration and escape of the input viral particle. Moreover, these molecular chaperones are co-opted to assist in the biogenesis and folding of new virus proteins which are essential for the formation of the new viral progenies.

In many instances, a virus simply uses a host chaperone’s well-established canonical function during entry. For example, the PDI oxidoreductase reduces and isomerizes the disulfide bonds in PyVs in the ER lumen to prime the virus for ER escape—PDI deploys this same activity on nascent cellular polypeptide chains as they are undergoing redox-dependent folding. Similarly, the ER-resident BiP promotes folding of the polyproteins of SARS-CoV-2, in much the same way BiP acts as a general chaperone to fold cellular clients. Establishing that viruses exploit the canonical activity of host chaperones provides a distinct advantage in developing rational antiviral therapies—any drugs that inhibit the normal functions of these chaperones can now be repurposed and tested to assess if they can likewise block virus infection. Future investigations along this line of research may prove useful.

By contrast, it is entirely possible that viruses exploit the noncanonical functions of host chaperones to support their infection. For instance, although γ-secretase is critical during HPV infection, the canonical proteolytic activity of this transmembrane protease—central to its normal cellular function—is not necessary during HPV entry. Instead, γ-secretase possesses a new “insertase” activity that promotes membrane insertion of HPV, enabling the virus to transport along a productive route [58]. This newly identified chaperone function of the γ-secretase raises the exciting possibility that its insertase activity might also be imparted to host proteins. As another example, cell surface attachment of the MERS-CoV and bCoV-HKU9 coronaviruses to BiP suggests that BiP—in addition to its well-established profolding chaperone activity—could act as a general recruitment factor for extracellular ligands at the plasma membrane. If so, BiP might bind to cellular ligands, potentially triggering their internalization and retrograde trafficking. 

In sum, these examples revealing unanticipated noncanonical functions of the host chaperones only further underscore the remarkable history of studying virus-host cell interaction. Not only do these studies provide critical insights into the basic mechanism of viral pathogenesis, they also expand the full repertoire of functions hidden in the host chaperones that might not have been revealed if not for these toxic pathogens.

## Data Availability

Not applicable.
